# EHS Rapid Guideline: Evidence-Informed European Recommendations on Parastomal Hernia Prevention—With ESCP and EAES Participation

**DOI:** 10.3389/jaws.2023.11549

**Published:** 2023-09-14

**Authors:** Cesare Stabilini, Filip E. Muysoms, Alexander A. Tzanis, Lisa Rossi, Ourania Koutsiouroumpa, Dimitris Mavridis, Michel Adamina, Umberto Bracale, Henk-Thijs Brandsma, Stéphanie O. Breukink, Manuel López Cano, Samantha Cole, Suzanne Doré, Kristian Kiim Jensen, Marianne Krogsgaard, Neil J. Smart, Christoffer Odensten, Chantal Tielemans, Stavros A. Antoniou

**Affiliations:** ^1^ Department of Surgery, University of Genoa, Genoa, Italy; ^2^ Department of Surgery, Maria Middelares Hospital, Ghent, Belgium; ^3^ Department of Medicine, University of Thessaly, Larissa, Greece; ^4^ Department of Surgery, IRCCS Policlinico San Martino, University of Genoa, Genoa, Italy; ^5^ Department of Primary Education, School of Education, University of Ioannina, Ioannina, Greece; ^6^ Department of Surgery, Cantonal Hospital Winterthur, Zurich, Switzerland; ^7^ Department of Public Health, University of Naples Federico II, Naples, Italy; ^8^ Department of Surgery, Antonius Ziekenhuis, Sneek, Netherlands; ^9^ Maastricht University Medical Centre, Maastricht, Netherlands; ^10^ Abdominal Wall Surgery Unit, Val d’ Hebrón University Hospital, Universidad Autónoma de Barcelona, Barcelona, Spain; ^11^ Patient Representative, Nottingham, United Kingdom; ^12^ Patient Representative, Rayne, United Kingdom; ^13^ Digestive Disease Center, Bispebjerg University Hospital, Copenhagen, Denmark; ^14^ Department of Surgery, Zealand University Hospital, Koege, Denmark; ^15^ Department of General Surgery, Royal Devon and Exeter Hospital, Exeter, United Kingdom; ^16^ Department of Surgical and Perioperative Sciences, Surgery, Umeå University Educational Unit at Sunderby Hospital, Sunderby, Sweden; ^17^ University Hospital Ghent, Ghent, Belgium; ^18^ Department of Surgery, Papageorgiou General Hospital, Thessaloniki, Greece

**Keywords:** stoma, ostomy, colostomy, mesh, prevention

## Abstract

**Background:** Growing evidence on the use of mesh as a prophylactic measure to prevent parastomal hernia and advances in guideline development methods prompted an update of a previous guideline on parastomal hernia prevention.

**Objective:** To develop evidence-based, trustworthy recommendations, informed by an interdisciplinary panel of stakeholders.

**Methods:** We updated a previous systematic review on the use of a prophylactic mesh for end colostomy, and we synthesized evidence using pairwise meta-analysis. A European panel of surgeons, stoma care nurses, and patients developed an evidence-to-decision framework in line with GRADE and Guidelines International Network standards, moderated by a certified guideline methodologist. The framework considered benefits and harms, the certainty of the evidence, patients’ preferences and values, cost and resources considerations, acceptability, equity and feasibility.

**Results:** The certainty of the evidence was moderate for parastomal hernia and low for major morbidity, surgery for parastomal hernia, and quality of life. There was unanimous consensus among panel members for a conditional recommendation for the use of a prophylactic mesh in patients with an end colostomy and fair life expectancy, and a strong recommendation for the use of a prophylactic mesh in patients at high risk to develop a parastomal hernia.

**Conclusion:** This rapid guideline provides evidence-informed, interdisciplinary recommendations on the use of prophylactic mesh in patients with an end colostomy. Further, it identifies research gaps, and discusses implications for stakeholders, including overcoming barriers to implementation and specific considerations regarding validity.

## Introduction

The incidence of parastomal hernia exceeds 50% in the long term, with substantial implications on patients’ quality of life [1, 2]. Reinforcement of the stoma with a mesh has been suggested to be associated with lower incidence of parastomal hernia [3].

A previous guideline of the European Hernia Society (EHS) issued a strong recommendation for the use of a synthetic permanent prophylactic mesh in the construction of an end colostomy [1]. This recommendation was primarily based upon evidence synthesis of randomized trials, suggesting a lower risk of parastomal hernia with the use of a prophylactic mesh without increasing perioperative and longer-term stoma-related complications [4–6].

The surgical literature has since seen a growing body of evidence on the use of synthetic permanent, absorbable, and biologic mesh for parastomal hernia prevention. In view of this new evidence and evolving methods in the field of clinical practice guidelines development, of an update of the guideline on parastomal hernias focused on prevention, and based upon an update systematic review, rigorous evidence appraisal, and a structured evidence-todecision framework informed by an international and interdisciplinary panel, including patient representatives. The objective is to inform clinical and patient decision making, and healthcare policy, to optimize the outcomes of stoma construction, and improve patients' quality of life.

## Methods

This rapid guideline follows AGREE-S, GRADE, Institute of Medicine, Guidelines International Network (GIN) and Cochrane Rapid Reviews Methods Group development and reporting standards [7–11]. It was registered at the International Practice Guideline Registry Platform (registration number IPGRP-2022CN216). An AGREE-S reporting checklist is provided in Supplementary File S2. We consulted GRADE official guidance published in a series of articles in the *Journal of Clinical Epidemiology* for up-to-date evidence appraisal and guideline development methodology. The development of this guideline was informed by the GRADE methodology to appraise the certainty of the evidence and the GRADE evidence-to-decision framework [12–15].

### Steering Group

The steering group consisted of 2 general and colorectal surgeons with specific interest in hernia surgery, members of the EHS Scientific Advisory Board (FM, CS), and a general surgeon, certified guideline methodologist and INGUIDE guideline certification credentialling instructor (SAA, INGUIDE certificate number 2021-L2-V1-00001). The guideline methodologist and a member of the steering group (FM) were coordinators of the previous EHS guideline on prevention and treatment of parastomal hernias [1]. The third member of the steering group (CS) acted as content coordinator of this project and disclosed no direct nor indirect conflict [16]. We therefore consider that a potential indirect conflict of the coordinators of the original guideline has not affected the content of this guideline.

### Guideline Panel

The guideline panel consisted of 3 colorectal surgeons (SB, NJS, MA), 2 general surgeons with specific interest in hernia surgery (KKJ, UB), 2 stoma care nurses (MK, CT), and 2 patient representatives (SC, SD). One of the patient representatives had an abdominoperineal resection 12 years ago for stage III colorectal cancer, and an end colostomy without prophylactic mesh. She developed a parastomal hernia months after surgery, and an incisional hernia at the site of an abdominal drain; she had not had any hernia repair. The other patient representative had an end colostomy construction without mesh for severe neurogenic bowel, slow transit constipation and passive fecal incontinence secondary to a spinal cord arteriovenous malformation. She had a revision surgery for a prolapse and possibly a small parastomal hernia with biological mesh reinforcement. At the time of the consensus meeting, she had no evident recurrence, apart from a subcutaneous prolapse. Both patient representatives were recruited through communication on Twitter.

The composition of panel members aimed to be representative of different parts of Europe and different age groups. Panel members disclosed no direct nor indirect conflicts [16]. We invited authors of randomized trials and evidence syntheses on the topic of interest as external advisors. These members were not involved in the decisions on the strength, the direction or the wording of the recommendations, but they were consulted in the development of the evidence-to-decision framework, as per GRADE and GIN guidance. The composition of the guideline development group and members’ roles are available in the online appendix [16].

### Health Question

Should prophylactic mesh versus no prophylactic mesh be used in patients who undergo construction of a permanent end colostomy?

This guideline refers to patients scheduled to undergo surgery that includes construction of a permanent end colostomy in the elective or emergency setting.

### Protocol

A protocol was developed *a priori* by the steering group [17]. The protocol draft was made publicly available through the social media, and the public was invited to comment on the content. The guideline question and outcomes of interest were refined in collaboration with the panel members. Amendments to the protocol with justifications are provided below.

### Rating the Importance of Outcomes

The importance of outcomes was rated by panel members using the GRADE scale [18]. The classification of outcomes into each of the three categories (not important, important, critical) was made by the steering group under consideration of panel members’ ratings available online (scale from 1 to 9, from the least to the highest importance) [16]. The final rating was the median of panel members’ ratings, since there was no substantial deviation from the median.

We considered the importance of outcomes as follows:

1. Clinical diagnosis of parastomal hernia: critical - 7
2. 30 days or in-hospital complications Clavien-Dindo ≥3: critical - 8
3. Surgery for parastomal hernia: critical - 8
4. Quality of life: critical - 9

The following outcomes were additionally prioritized by the panel: pain, size of bulge, spillage, time off work, and computed tomography-classified diagnosis. The steering group considered that the outcomes pain, size of bulge, time off work, and spillage are overlapping with the outcomes: clinical diagnosis, 30 days or in-hospital complications, and quality of life. Computed tomography-classified diagnosis was not prioritized, because it was considered a non-patient-important outcome. An external advisor suggested that imaging findings of a parastomal hernia not evident on clinical examination may predict the development of a clinical parastomal hernia. Relevant evidence was, therefore, taken into account, albeit not in summary analyses.

Furthermore, the panel was informed that the outcomes clinical diagnosis of parastomal hernia, surgery for parastomal hernia, and quality of life may overlap to a substantial extent; they were, therefore, advised to exercise caution in their judgments on overall benefit/harm in the context of the evidence-to-decision framework.

### Definitions

We considered clinical diagnosis of parastomal hernia negatively affecting patient experience as the outcome of interest of this guideline. The studies providing the evidence base of this work provided different definitions of clinically evident (but not necessarily clinically relevant) parastomal hernia, but we did not consider justified to downgrade for indirectness (different definition of the outcome in the source studies versus the definition used in this guideline). The definitions of parastomal hernia, clinical and/or radiological, used by each author group are provided in the online appendix [16]. Furthermore, we considered the pooled comparative effect estimates of clinical and clinical/radiological diagnosis of parastomal hernia, because sensitivity analyses suggested similar *comparative effect estimates* between clinical versus clinical/radiological diagnosis of parastomal hernia (see statistical analyses in the online appendix [16]).

### Setting Minimal Important Differences

The evidence-to-decision framework was set within a fully contextualized approach [19]. This approach considers all relevant outcomes for clinical decision making and entails setting a decision threshold for clinically meaningful effect for each outcome. An anonymous web-based survey of panel members was performed to define decision thresholds (minimal important differences). The results of the survey are available online [16]. The final rating was the median of panel members’ ratings, since no substantial deviations from the median were observed.

Under consideration of panel’s responses, the following minimal important differences were set:

1. Clinical diagnosis of parastomal hernia: 50 per 1,000 patients
2. 30-day or in-hospital complications Clavien-Dindo ≥3: 50 per 1,000 patients
3. Surgery for parastomal hernia: 50 per 1,000 patients
4. Quality of life: 25 out of 100 points, or 0.2/0.5 standard deviation units (small/moderate difference)

The outcome quality of life was reported under different scales (EORTC QLQ-C30, Short Form 36, Stoma QoL questionnaire); we therefore calculated standardized mean differences. Although no universal cutoff can be applied [20], we considered the above differences in standard deviation units as important for small/moderate difference, based on expert guidance (INGUIDE McMaster guideline methodologist certification program).

### Systematic Review and Evidence Synthesis

The full systematic review with details on the search strategy, study selection, data extraction, risk of bias assessment, statistical analyses (including sensitivity analyses), and assessment of the certainty of the evidence is published separately in this issue [21].

In brief, we updated a previous systematic review with *de novo* evidence search of PubMed. Study selection, data extraction, and risk of bias assessment were performed by an *ad hoc* evidence research team (AT, LR), and statistical analyses were performed independently by a statisticians’ team. We considered randomized controlled trials only, comparing the use of prophylactic mesh versus no mesh in the construction of an end colostomy. Overarching inclusion criteria were adult patients undergoing surgery with construction of an end colostomy for either benign or malignant disease, in an elective or emergency setting. An external advisor (HTB) provided long-term data of the PREVENT trial, which were unpublished by the time of development of the evidence tables, but were published recently [22]. Therefore, we do not consider that the risk of including these data was high. Another two external advisors indicated that longer-term data of their trials have been collected, but they were not available for third-party use.

We performed *de novo* risk of bias assessments using RoB-2 [23]. For the purposes of outcome-specific risk of bias assessment, outcomes were grouped as follows: 30 days complications Clavien-Dindo ≥3; parastomal hernia and surgery for parastomal hernia; and quality of life. We considered longest-term follow-up data for all outcomes, with a minimum follow-up of 12 months, except for perioperative complications. The panel considered 5-year follow-up as sufficient in the context of this guideline (median of votes).

We conducted random and fixed effect(s) meta-analyses to synthesize evidence. We used the DerSimonian & Laird estimator for the between study-variance. For the continuous outcome, we extracted the mean, the sample size and the standard deviation, and we estimated the study-specific standardized mean differences along with the corresponding 95% confidence interval for each group. For what we could not calculate the standard deviation, we used the maximum standard deviation among studies. We explored heterogeneity via the I^2^ statistic and by computing the Q-statistic and the 95% predictive intervals. We performed sensitivity analyses of studies with a minimum follow-up duration of 5 years and compared the effect estimates with studies with shorter follow-up duration. We also performed subgroup analyses based on the anatomical position of the mesh (retrorectus/intraperitoneal/anterectus). We did not observe subgroup differences; we therefore considered the overall effect estimate.

### Assessment of the Certainty of Evidence

We constructed GRADE evidence profiles of certainty for each pairwise comparison separately and for each outcome using GRADEpro GDT [24]. The certainty of evidence is determined by the risk of bias across studies, incoherence, indirectness, imprecision, publication bias and other parameters [25]. To inform calculations of absolute effect differences, we performed proportion meta-analyses of frequencies of baseline risks/effects provided by the source studies; these are available in the online appendix [16]. One study only provided data to allow time-to-event analyses [26], therefore time-to-event data meta-analysis could not be performed.

### Evidence-to-Decision Framework and Development of Recommendations

We provided the evidence tables to the guideline panel for review in advance of an in-person consensus meeting. In the consensus meeting, the guideline development methodology was detailed, and panel members provided their judgements on:

• the magnitude of benefit of the intervention compared to the control
• the magnitude of harm of the intervention compared to the control
• the certainty of the evidence on benefits and harms
• any variability in patients’ values and preferences
• costs or savings related to the intervention compared to the control
• effect of the intervention on equity compared to the control
• acceptability of the intervention compared to the control
• feasibility of the intervention compared to the control
Panel members then participated in an online Delphi process to formulate the recommendation. A draft of the recommendation was developed by the steering group, and panel members were invited to anonymously propose modifications.

### Amendments to the Protocol

For logistical reasons, we included 2 general surgeons with specific interest in hernia surgery, instead of 3. We searched PubMed, instead of MEDLINE via the Healthcare Databases Advanced Search interface, because the latter was not available since March 2022. OpenGrey was neither operational by the time of the update search.

## Results

We identified 19 reports on 12 unique randomized trials [3, 22, 26–42] (PRISMA 2020 flow chart available in the online appendix [16]). Ten trials reported on elective surgery, 11 trials reported primarily on patients with malignancy as background pathology that necessitated construction of a stoma, and all trials reported on the use of synthetic non-absorbable or partially absorbable mesh. Detailed study characteristics are provided in the data extraction sheet available in the online appendix [16]. Detailed statistical analyses are available in the online appendix [16].

The evidence profile is provided in Table 1 and in Table 2 as summary of findings. Table 3 details the evidence-to-decision considerations.

**TABLE 1:** Evidence summary on the use of mesh for parastomal hernia prevention.

Certainty assessment							No. of patients		Effect		Certainty	Importance
No. of studies	Study design	Risk of bias	Inconsistency	Indirectness	Imprecision	Other considerations	Prophylactic mesh	No prophylactic mesh	Relative (95% CI)	Absolute (95% CI)		
Major morbidity (30 day) (assessed with: Clavien-Dindo ≥3)												
2^a^	Randomised trials	Not serious	Not serious	Not serious	Very serious^b^	None	30/135 (22.2%)	7.3%	OR 0.77 (0.45–1.30)	16 fewer per 1,000 (from 39 fewer to 20 more)	⊕⊕○○ Low	CRITICAL
								23.8%		44 fewer per 1,000 (from 115 fewer to 51 more)		
								55.3%		65 fewer per 1,000 (from 195 fewer to 64 more)		
Parastomal hernia (follow-up: mean 5 years; assessed with: physical examination)												
12	Randomised trials	Not serious^c^	Serious^d^	Not serious^e^	Not serious	None^f^	123/457 (26.9%)	36.3%	OR 0.33 (0.18–0.62)	205 fewer per 1,000 (from 270 fewer to 102 fewer)	⊕⊕⊕○ Moderate	CRITICAL
								45.3%		238 fewer per 1,000 (from 323 fewer to 114 fewer)		
								54.7%		262 fewer per 1,000 (from 368 fewer to 119 fewer)		
Surgery for parastomal hernia (follow-up: mean 5 years)												
3	Randomised trials	Serious^g^	Not serious	Not serious^h^	Serious^i^	None	3/115 (2.6%)	2.5%	OR 0.18 (0.06–0.59)	20 fewer per 1,000 (from 23 fewer to 10 fewer)	⊕⊕○○ Low	CRITICAL
								5.0%		41 fewer per 1,000 (from 47 fewer to 20 fewer)		
								9.5%		76 fewer per 1,000 (from 89 fewer to 37 fewer)		
Quality of life (follow-up: range 1 year to 5 years; assessed with: EORTC QLQ-C30, Short Form 36, Stoma QoL questionnaire)												
3	Randomised trials	Very serious^j^	Not serious	Not serious	Not serious	None	266	267	—	SMD 0.03 SD higher (0.14 lower to 0.2 higher)	⊕⊕○○ Low	CRITICAL

CI, confidence interval; OR, odds ratio; SMD, standardised mean difference.

Explanations.

^a^ The top row in each set of absolute effect estimates represents estimated difference in low baseline risk patients, the middle row represents estimated difference in moderate baseline risk patients, and the bottom row represents estimated difference in high baseline risk patients.

^b^ Very wide confidence interval crossing lower and upper decision thresholds, unless low baseline risk of major morbidity.

^c^ Several studies with some concerns. Sensitivity (random effects) and meta-regression analysis did not indicate substantially different effect estimates of studies at low risk of bias versus those with some concerns (see sensitivity analyses in online appendix). Therefore, we did not downgrade the certainty of evidence in this domain.

^d^ Substantial heterogeneity (I2 = 73%), however we did not downgrade for both heterogeneity and imprecision, because the former is mitigated by the random effects model and is addressed by the domain of imprecision.

^e^ Sensitivity (random effects) and meta-regression analysis did not indicate substantial difference between studies with follow-up ≥5 years versus <5 years (panel-set threshold for minimal clinical importance). We therefore considered the pooled comparative outcome irrespective of duration of follow-up.

^f^ Asymmetrical funnel plot and significant evidence of publication bias on Egger’s test (*p* = 0.0002) in summary analysis; however, we did not double-downgrade for both heterogeneity and publication bias, because of overlapping effects.

^g^ Several studies with some concerns. Sensitivity (random effects) and meta-regression analysis did not indicate substantially different effect estimates of studies at low risk of bias versus those with some concerns (see sensitivity analyses in online appendix). However, visual inspection of sensitivity analyses suggest that there may be inflated effect estimates in studies with some concerns/high risk, that is statistically undetectable. Therefore, we downgraded the certainty of evidence in this domain by one level.

^h^ Sensitivity (random effects) and meta-regression analysis indicated substantial difference between studies with follow-up ≥5 years versus <5 years. We therefore considered studies reporting ≥5 years data only (panel-set threshold for minimal clinical importance).

^i^ Downgraded due to few events, and because the confidence interval is crossing lower decision threshold when highest baseline risk is considered.

^j^ Mostly due to missing data.

**TABLE 2:** Summary of findings table on the use of mesh for parastomal hernia prevention.

Prophylactic mesh compared to no prophylactic mesh in patients who undergo construction of a permanent end colostomy						
Patient or population: patients who undergo construction of a permanent end colostomy						
Setting: healthcare/Europe						
Intervention: prophylactic mesh						
Comparison: no prophylactic mesh						
Outcomes	Anticipated absolute effects^a^ (95% CI)		Relative effect (95% CI)	No of participants (studies)	Certainty of the evidence (GRADE)	Comments
	Risk with no prophylactic mesh	Risk with prophylactic mesh				
Major morbidity (30 days) (Major morbidity) assessed with: Clavien-Dindo ≥3	Low		OR 0.77 (0.45–1.30)	359 (2 RCTs)^b^	⊕⊕○○ Low^c^	Prophylactic mesh may result in little to no difference in the risk of major morbidity in the elective setting
	73 per 1,000	57 per 1,000 (34–93)				
	Moderate					
	238 per 1,000	194 per 1,000 (123–289)				
	High					
	553 per 1,000	488 per 1,000 (358–617)				
Parastomal hernia (PSH) assessed with: physical examination follow-up: mean 5 years	Low		OR 0.33 (0.18–0.62)	997 (12 RCTs)	⊕⊕⊕○ Moderate^d^ ^,^ ^e^ ^,^ ^f^ ^,^ ^g^	Prophylactic mesh likely results in a reduction in parastomal hernia
	363 per 1,000	158 per 1,000 (93–261)				
	Moderate					
	453 per 1,000	215 per 1,000 (130–339)				
	High					
	547 per 1,000	285 per 1,000 (179–428)				
Surgery for parastomal hernia (Surgery for PSH) follow-up: mean 5 years	Low		OR 0.18 (0.06–0.59)	234 (3 RCTs)	⊕⊕○○ Low^h^ ^,^ ^i^ ^,^ ^j^	Prophylactic mesh may result in little to no difference in surgery for parastomal hernia
	25 per 1,000	5 per 1,000 (2–15)				
	Moderate					
	50 per 1,000	9 per 1,000 (3–30)				
	High					
	95 per 1,000	19 per 1,000 (6–58)				
Quality of life (QoL) assessed with: EORTC QLQ-C30, Short Form 36, Stoma QoL questionnaire follow-up: range 1 year–5 years	—	SMD 0.03 SD higher (0.14 lower to 0.2 higher)	—	533 (3 RCTs)	⊕⊕○○ Low^k^	Prophylactic mesh may result in little to no difference in quality of life

CI, confidence interval; OR, odds ratio; SMD, standardised mean difference.

GRADE Working Group grades of evidence.

High certainty: we are very confident that the true effect lies close to that of the estimate of the effect.

Moderate certainty: we are moderately confident in the effect estimate: the true effect is likely to be close to the estimate of the effect, but there is a possibility that it is substantially different.

Low certainty: our confidence in the effect estimate is limited: the true effect may be substantially different from the estimate of the effect.

Very low certainty: we have very little confidence in the effect estimate: the true effect is likely to be substantially different from the estimate of effect.

Explanations.

^a^ The risk in the intervention group (and its 95% confidence interval) is based on the assumed risk in the comparison group and the relative effect of the intervention (and its 95% CI).

^b^ The top row in each set of absolute effect estimates represents estimated difference in low baseline risk patients, the middle row represents estimated difference in moderate baseline risk patients, and the bottom row represents estimated difference in high baseline risk patients.

^c^ Very wide confidence interval crossing lower and upper decision thresholds, unless low baseline risk of major morbidity.

^d^ Several studies with some concerns. Sensitivity (random effects) and meta-regression analysis did not indicate substantially different effect estimates of studies at low risk of bias versus those with some concerns (see sensitivity analyses in online appendix). Therefore, we did not downgrade the certainty of evidence in this domain.

^e^ Substantial heterogeneity (I2 = 73%), however we did not downgrade for both heterogeneity and imprecision, because the former is mitigated by the random effects model and is addressed by the domain of imprecision.

^f^ Sensitivity (random effects) and meta-regression analysis did not indicate substantial difference between studies with follow-up ≥5 years versus <5 years (panel-set threshold for minimal clinical importance). We therefore considered the pooled comparative outcome irrespective of duration of follow-up.

^g^ Asymmetrical funnel plot and significant evidence of publication bias on Egger’s test (*p* = 0.0002) in summary analysis; however, we did not double-downgrade for both heterogeneity and publication bias, because of overlapping effects.

^h^ Several studies with some concerns. Sensitivity (random effects) and meta-regression analysis did not indicate substantially different effect estimates of studies at low risk of bias versus those with some concerns (see sensitivity analyses in online appendix). However, visual inspection of sensitivity analyses suggest that there may be inflated effect estimates in studies with some concerns/high risk, that is statistically undetectable. Therefore, we downgraded the certainty of evidence in this domain by one level.

^i^ Sensitivity (random effects) and meta-regression analysis indicated substantial difference between studies with follow-up ≥5 years versus <5 years. We therefore considered studies reporting ≥5 years data only (panel-set threshold for minimal clinical importance).

^j^ Downgraded due to few events, and because the confidence interval is crossing lower decision threshold when highest baseline risk is considered.

^k^ Mostly due to missing data.

**TABLE 3:** Evidence-to-decision framework on the use of mesh for parastomal hernia prevention.

A) Question		
Should prophylactic mesh vs. no prophylactic mesh be used for patients who undergo construction of a permanent end colostomy?
Population:	Patients who undergo construction of a permanent end colostomy
Intervention:	prophylactic mesh
Comparison:	no prophylactic mesh
Main outcomes:	Major morbidity (30 day); Parastomal hernia; Surgery for parastomal hernia; Quality of life
Setting:	healthcare/Europe

B) Assessment		
Problem		
Is the problem a priority?		

Judgement	Research evidence	Additional considerations
○ No	Evidence suggests that parastomal hernia substantially affects patients’ quality of life [2].	
○ Probably no		
○ Probably yes		
• Yes	The healthcare question was prioritized by the European Hernia Society in view of ongoing debate about the relative effectiveness of prophylactic mesh for the construction of end colostomy and new evidence since the publication of previous guidelines. It was also prioritized in a members’ survery of the Association of Coloproctology of Great Britaine and Ireland [43] and a survey of the members of the American Society of Colon and Rectal Surgeons [44].	
○ Varies		
○ Don’t know		
Desirable effects		
How substantial are the desirable anticipated effects?		
○ Trivial	Evidence demonstrates that mesh prophylaxis likely results in a reduction in parastomal hernia, and may result in little to no difference in the risk for surgery for parastomal hernia. There may not be any difference in effects compared to no mesh with regards to major complications, surgery for parastomal hernia and quality of life.	Surgery for parastomal hernia is an individual decision influenced by patients’ and surgeons’ decisions. Considering study demographics, the evidence summarized herein probably reflects practice variation in the wider European region.
○ Small		
• Moderate		
○ Large	Sensitivity analyses excluding the unpublished data of the PREVENT trial and the data of the PARTHENOPE trial on Bio-A mesh did not suggest different effects. Detailed statistical analyses are available in the online appendix.	
○ Varies		
○ Don’t know		
Undesirable effects		
How substantial are the undesirable anticipated effects?		
○ Large	No substantial evidence on undesirable effects was found. A scoping search on PubMed with the search syntax (mesh erosion) AND (*stomy OR stoma) did not suggest that mesh erosion is a pragmatic risk after parastomal hernia prevention with a prophylactic mesh. Randomized trials may not be the best study design to capture rare events. We performed an additional scoping search of observational studies (available in the online appendix), that did not identify any reported cases of mesh erosion with the use of a prophylactic mesh, albeit with mean/median follow-up duration between 11 and 60 months for 242 patients.	A minority of panel members suggested that undesirable effects were small rather than trivial. Harms related to the use of prophylactic mesh, (such as erosion, infection, stenosis) may exist, although published evidence does not report any substantial risk for harm. Under consideration of current published evidence, it is unlikely that the burden of any potential harm is substantial.
○ Moderate		
○ Small		
• Trivial		
○ Varies		
○ Don’t know		
Certainty of evidence		
What is the overall certainty of the evidence of effects?		
○ Very low	The certainty of evidence was low or moderate for outcomes of critical importance, therefore the overall certainty of evidence was considered to be low to moderate.	The panel considered that the certainty of the evidence is sufficient for most outcomes, including parastomal hernia, major morbidity and reoperation. However, quality of life is underreported, which does not allow for sufficient overall certainty on critical outcomes. There is no reason to suspect that patients with prophylactic mesh have a poorer quality of life compared to patients with mesh; nevertheless, it is crucial to collect additional evidence before supporting a strong recommendation.
• Low		
○ Moderate		
○ High		
○ No included studies		
Values		
Is there important uncertainty about or variability in how much people value the main outcomes?		
• Important uncertainty or variability	Research suggests that parastomal hernia frequently affects patients’ quality of life to a substantial degree [2]. However, in a scoping search, no research was found that has focused on the value patients place on outcomes after construction of an end colostomy.	After we have presented the summary evidence in interactive form on GRADEpro to patient representatives, both agreed that the vast majority of patients would opt for prophylactic mesh.
○ Possibly important uncertainty or variability		One of the patient representatives, who had an end colostomy for cancer, highlighted that it might be difficult for the patient to handle much information in addition to that related to their disease, the operation, the postoperative course and adjuvant therapy.
○ Probably no important uncertainty or variability		The other patient representative, who was operated on for benign disease and was a medical professional, preferred being provided with sufficient information to decide on the intervention.
○ No important uncertainty or variability	We do not anticipate that there would be substantial variability on the value patients place on quality of life, parastomal hernia, major morbidity and reoperation.	Both patient representatives reported that they would be substantially influenced by the opinion and preferences of their surgeon. Empirical evidence and the ongoing debate in surgical journals and social media, suggests that there is important variability in surgeons’ opinions and preferences. Therefore, it may be assumed that this variability will be reflected on patient decisions.
		In addition, it was suggested that some older patients might not prefer a prophylactic mesh, whereas younger patients operated on for benign disease would prefer the intervention
		Furthermore, it was noted that patients with specific values and beliefs would want to be informed about the material of a biological mesh.
Balance of effects		
Does the balance between desirable and undesirable effects favor the intervention or the comparison?		
○ Favors the comparison	There was unanimous agreement that the balance of effects was in favor of prophylactic mesh.	With regards to the lack of difference between the intervention and the comparator in the effects on quality of life, it was suggested that, because the evidence was derived primarily from patients with cancer, the primary disease may dominate patients’ experience and their own-perception on quality of life.
○ Probably favors the comparison		
○ Does not favor either the intervention or the comparison		
• Probably favors the intervention		
○ Favors the intervention		
○ Varies		
○ Don’t know		
Resources required		
How large are the resource requirements (costs)?		
○ Large costs	Under consideration of a cost analysis [46] and a cost-effectiveness analysis [47], that takes into account the cost of the mesh, evidence suggests that prophylactic mesh results in substantial savings.	The intervention does not require additional resources with regards to personnel, and only moderate additional resources with regards to operation time, based on empirical evidence.
○ Moderate costs ○ Negligible costs and savings• Moderate savings○ Large savings○ Varies○ Don’t know	No additional operating time was suggested by a meta-analysis of randomized trials [46].
○ Don’t know
Certainty of evidence of required resources		
What is the certainty of the evidence of resource requirements (costs)?		
○ Very low	The quality of relevant research is at least moderate.	
○ Low		
• Moderate		
○ High		
○ No included studies		
Cost effectiveness		
Does the cost-effectiveness of the intervention favor the intervention or the comparison?		
○ Favors the comparison	“Synthetic mesh was less costly and more effective than biologic and no mesh to prevent PSH for all rectal cancer stages. At the willingness-to-pay threshold of £20,000/QALY, the incremental NMBs [95% CI] ranged between £3,412 [£3,384–£3,439] (stage-I) and £1,305 [£1,293–£1,316] (stage-IV) for synthetic vs. no mesh. Synthetic mesh was more cost-effective than no mesh unless the relative risk of PSH was ≥0.97 for stages I–III and ≥0.94 for stage-IV.” [47].	
○ Probably favors the comparison		
○ Does not favor either the intervention or the comparison		
○ Probably favors the intervention		
• Favors the intervention		
○ Varies		
○ No included studies		
Equity		
What would be the impact on health equity?		
○ Reduced	No relevant evidence found.	The panel did not identify any substantial impact on equity. The additional use of operating room time was considered negligible. No special skills were thought to be required for the implementation on the intervention. The low cost of synthetic mesh also suggests no impact on equity.
○ Probably reduced		
• Probably no impact		
○ Probably increased		
○ Increased		
○ Varies		
○ Don’t know		
Acceptability		
Is the intervention acceptable to key stakeholders?		
○ No	Published [36, 48–53] and empirical evidence suggests that acceptability varies among surgeons.	The ongoing debate in surgical journals, social media, and empirical evidence, suggests that the intervention might not be acceptable to a substantial proportion of surgeons.
○ Probably no		Furthermore, creation of the stoma is frequently been performed by trainees or junior surgeons, and the primary surgeon might not always oversee this part of the procedure.
○ Probably yes		There were no concerns with regards to the acceptability of the intervention to patients and stoma care nurses.
○ Yes• Varies○ Don’t know	No systematically collected published data were found on surgeons, patients and other stakeholders.	
Feasibility		
Is the intervention feasible to implement?		
○ No	No relevant evidence identified.	The panel considered that the intervention is feasible to be performed, with no substantial challenges with regards to surgical technique, however some surgeons might need minimal training before performing the intervention.
○ Probably no		
• Probably yes		
○ Yes		
○ Varies		
○ Don’t know		

There was unanimous consensus on the direction, the strength, and the wording of the recommendations within the first Delphi round [16].

### Recommendations

 • We suggest the use of a synthetic non-absorbable prophylactic mesh for the construction of an end colostomy.

Conditional recommendation

 • We recommend the use of a synthetic non-absorbable prophylactic mesh for the construction of an end colostomy in patients at high risk for parastomal hernia (patients with a history of an abdominal wall hernia, connective tissue disorder, obesity, likely to undergo chemotherapy) and life expectancy over 2 years.

Strong recommendation.

## Discussion

### Implications for Policy Makers

Policymakers are called to facilitate a parastomal hernia prevention strategy for most patients. This includes availability of synthetic mesh and additional operating room time. Training of surgeons to perform stoma construction with a prophylactic mesh may be necessary in some settings.

### Implications for Healthcare Professionals

General and colorectal surgeons are called to discuss management options with their patients, providing detailed relative probabilities of the occurrence of key outcomes (see GRADE evidence table and Supplementary File S1, S2). When informed appropriately, the majority of patients is expected to opt for a parastomal hernia prevention with a synthetic non-absorbable mesh.

The clinical diagnosis of parastomal hernia, which was a core outcome in this guideline, may not always be important for patients (for example, small clinically detectable hernias, or hernias not causing symptoms and not affecting quality of life), and important variability may be expected in this regard.

The users of this guideline must be aware that the evidence informing these recommendations derives primarily, but not exclusively, from trials on elective surgery. Most trial reports did not provide subgroup data on emergency and elective surgery or clean and contaminated surgery, that would inform recommendations on these patient subgroups in the best possible way.

Furthermore, other factors, such as smoking and radiotherapy, might also be risk factors for the development of a parastomal hernia, whereas some of the listed factors might have a lesser effect. Findings from the CIPHER study are anticipated to provide more precise estimates on the effect of various factors on the risk of parastomal hernia, and users are advised to stay informed by the most recent evidence (see also *Author Disclaimer*).

### Implications for Patients

Patients can be informed that the risks of perioperative complications, surgery for parastomal hernia and quality of life may be similar between prophylactic mesh versus no mesh. However, mesh prophylaxis likely reduces the risk of parastomal hernia by more than 50% (from 45% without mesh to 22% with mesh). There are no known frequent adverse events related to the mesh.

Of note, recent pooled long-term data from 3 randomized trials suggests that prophylactic mesh delays the occurrence of a parastomal hernia by about 5 years, rather than prevents it overall [45]. This hypothesis could not be tested across studies in the present analysis, because none had provided Kaplan-Meier plots nor hazard ratios, that would allow for time-to-event meta-analyses.

### Implications for Researchers

The following research gaps were identified.

Randomized trials:

 ‐ Long-term quality of life (low certainty)

 ‐ Perioperative morbidity; ideally classified using the Clavien-Dindo classification (low certainty)

 ‐ Surgery for parastomal hernia (low certainty)

 ‐ Subgroup data on emergency/elective surgery, and contaminated/clean-contaminated surgery

 ‐ Reporting both clinical and radiological recurrence. Significant clinical recurrence may be important to patients, however, radiological recurrence may predict future clinical recurrence.

Observational studies:

 ‐ Long-term morbidity

Survey or qualitative studies:

 ‐ Patients’, surgeons’ and stoma care nurses’ values and preferences related to the main outcomes

 ‐ Patients’, surgeons’ and stoma care nurses’ thresholds for small, moderate and large effects with regards to key outcomes

 ‐ Core outcome set of patient-important outcomes for studies reporting on stomas

### Barriers and Facilitators

Implementing the intervention might be the most important challenge for surgeons who have not relevant experience. The majority of published information is derived from studies where a retromuscular mesh reinforcement was applied, and this practice may be particularly challenging to surgeons who are not familiar with abdominal wall reconstruction. We advise clinical visits or fellowships if the surgeon is not confident in using a prophylactic mesh. Professional organizations might want to consider offering training courses on cadaveric or animal models.

Organizational culture and resistance to change might be a substantial barrier. This document and the associated systematic review and meta-analysis [21] should serve as an independent, reliable source of information, that provides solid evidence on the balance between benefits and harms, in favor of benefits, especially from the patients’ perspective. Open, evidence-informed discussions and review of the evidence contained herein may inform organizational policy in the best possible way.

### Monitoring

Use of the guideline by EHS members will be monitored through an online survey 2 years after publication. Feedback from target users in the form of email communications, letters to the editor, and comments in social media will be documented to be addressed in future versions of this guideline.

We suggest auditing of outcomes of stoma construction with a mesh and comparison to international standards, with observed risk intervals (based upon proportion meta-analysis of risks and 95% confidence intervals in the source studies; see online appendix [16]):

 • Parastomal hernia: 21.1% (13.8%–31.0%)
 • Major complications (Clavien-Dindo ≥3): 20.2% (7.2%–45.3%)

### Validity Period

A scoping search of clinicaltrials.gov in March 2023 using the syntax (*stoma OR ostomy OR colostomy*) *AND mesh* with no limitations identified 47 records. Only one trial was identified as ongoing (NCT03799939), with estimated completion date in 2026. An average of 1.2 reports of randomized trials were published per year between 2012 and 2021. Substantial change in intervention effects and/or additional data on underreported outcomes (quality of life, surgery for parastomal hernia) is not expected earlier than 6 years since the last search. These recommendations are valid until December 2028.

### Update

This guideline is planned to be updated in 2028, unless substantial new evidence will be identified.

## Conclusion

A European interdisciplinary panel including patient representatives suggests the use of a synthetic non-absorbable mesh when constructing an end colostomy and recommends the routine use of a synthetic non-absorbable mesh in high-risk patients.

## Data Availability

The datasets presented in this study can be found in online repositories. The names of the repository/repositories and accession number(s) can be found below: https://osf.io/k4sh8/.

## Author Disclaimer

This clinical practice guideline has been developed under the auspice of the European Hernia Society (EHS). It is intended to be used primarily by health professionals (e.g., surgeons, anaesthetists, physicians) and to assist in making informed clinical decisions on diagnostic measures and therapeutic management. It is also intended to inform individual practice of allied health professionals (e.g., surgical nurses, dieticians, physical rehabilitation therapists, psychologists); to inform strategic planning and resource management by healthcare authorities (e.g., regional and national authorities, healthcare institutions, hospital administration authorities); and to inform patients wishing to obtain an overview of the condition of interest and its management. The use of recommendations contained herein must be informed by supporting evidence accompanying each recommendation and by research evidence that might not have been published by the time of writing the present document. Users must thus base their actions informed by newly published evidence at any given point in time. The information in the guideline should not be relied upon as being complete or accurate, nor should it be considered as inclusive of all proper treatments or methods of care or as a statement of the standard of care. With the rapid development of scientific knowledge, new evidence may emerge between the time the guideline is developed and when it is published or read. The guideline is not continually updated and may not reflect the most recent evidence. The guideline addresses only the topics specifically identified therein and is not applicable to other interventions, diseases, or stages of diseases. This guideline does not mandate any particular course of medical care. Further, the guideline is not intended to substitute the independent professional judgment of the treating provider, as the guideline does not necessarily account for individual variation among patients. Even if evidence on a topic suggests a specific diagnostic and/or treatment action, users and especially health professionals may need to decide against the suggested or recommended action in view of circumstances related to patient values, preferences, co-morbidities and disease characteristics; available human, monetary and material resources; and healthcare infrastructures. EHS provides this guideline on an “as is” basis, and makes no warranty, express or implied, regarding the guideline.

## References

[1] Antoniou SA Agresta F Garcia Alamino JM Berger D Berrevoet F Brandsma HT European Hernia Society Guidelines on Prevention and Treatment of Parastomal Hernias. Hernia (2017) 22:183–98. 10.1007/s10029-017-1697-5 29134456

[2] van Dijk SM Timmermans L Deerenberg EB Lamme B Kleinrensink GJ Jeekel J Parastomal Hernia: Impact on Quality of Life? World J Surg (2015) 39(10):2595–601. 10.1007/s00268-015-3107-4 26216640

[3] Jänes A Cengiz Y Israelsson LA. Randomized Clinical Trial of the Use of a Prosthetic Mesh to Prevent Parastomal Hernia. Br J Surg (2004) 91(3):280–2. 10.1002/bjs.4417 14991626

[4] Shabbir J Chaudhary BN Dawson R. A Systematic Review on the Use of Prophylactic Mesh During Primary Stoma Formation to Prevent Parastomal Hernia Formation. Colorectal Dis (2012) 14(8):931–6. 10.1111/j.1463-1318.2011.02835.x 21929523

[5] Tam KW Wei PL Kuo LJ Wu CH. Systematic Review of the Use of a Mesh to Prevent Parastomal Hernia. World J Surg (2010) 34(11):2723–9. PMID: 20661562. 10.1007/s00268-010-0739-2 20661562

[6] Sajid MS Kalra L Hutson K Sains P. Parastomal Hernia as a Consequence of Colorectal Cancer Resections Can Prophylactically Be Controlled by Mesh Insertion at the Time of Primary Surgery: A Literature Based Systematic Review of Published Trials. Minerva Chir (2012) 67(4):289–96. PMID: 23022753. 23022753

[7] Schünemann H Brożek J Guyatt G Oxman A. GRADE Handbook for Grading Quality of Evidence and Strength of Recommendations (2013). Updated October 2013. Available from: https://gdt.gradepro.org/app/handbook/handbook.html (Accessed January 1, 2022).

[8] AGREE. AGREE-S: AGREE II Extension for Guidelines on Surgical Interventions (2022). Available from: https://agree-s.org/ (Accessed January 1, 2022).

[9] Institute of Medicine (US). Institute of Medicine (US) Committee on Standards for Developing Trustworthy Clinical Practice Guidelines: Clinical Practice Guidelines We Can Trust (2011).

[10] Qaseem A Forland F Macbeth F Ollenschläger G Phillips S van der Wees P Guidelines International Network: Toward International Standards for Clinical Practice Guidelines. Ann Intern Med (2012) 156(7):525–31. PMID: 22473437. 10.7326/0003-4819-156-7-201204030-00009 22473437

[11] Garritty C Gartlehner G Nussbaumer-Streit B King VJ Hamel C Kamel C Cochrane Rapid Reviews Methods Group Offers Evidence-Informed Guidance to Conduct Rapid Reviews. J Clin Epidemiol (2021) 130:13–22. Epub 2020 Oct 15. PMID: 33068715; PMCID: PMC7557165. 10.1016/j.jclinepi.2020.10.007 33068715 PMC7557165

[12] Schünemann H Broże J Guyatt G. GRADE Handbook: 5 Quality of Evidence (2022). Available from: https://gdt.gradepro.org/app/handbook/handbook.html#h.9rdbelsnu4iy (Accessed June 19, 2022).

[13] Guyatt G Oxman AD Sultan S Brozek J Glasziou P Alonso-Coello P GRADE Guidelines: 11. Making an Overall Rating of Confidence in Effect Estimates for a Single Outcome and for All Outcomes. J Clin Epidemiol (2013) 66(2):151–7. Epub 2012 Apr 27. PMID: 22542023. 10.1016/j.jclinepi.2012.01.006 22542023

[14] Andrews JC Schünemann HJ Oxman AD Pottie K Meerpohl JJ Coello PA GRADE Guidelines: 15. Going from Evidence to Recommendation-Determinants of a Recommendation's Direction and Strength. J Clin Epidemiol (2013) 66(7):726–35. Epub 2013 Apr 6. PMID: 23570745. 10.1016/j.jclinepi.2013.02.003 23570745

[15] Andrews J Guyatt G Oxman AD Alderson P Dahm P Falck-Ytter Y GRADE Guidelines: 14. Going from Evidence to Recommendations: The Significance and Presentation of Recommendations. J Clin Epidemiol (2013) 66(7):719–25. Epub 2013 Jan 9. PMID: 23312392. 10.1016/j.jclinepi.2012.03.013 23312392

[16] Antoniou SA. Appendix Files for EHS Rapid Guideline: Update Systematic Review, Meta-Analysis GRADE Assessment, and Evidence-Informed European Recommendations on Parastomal Hernia Prevention – with ESCP and EAES Participation (2022). Available from: https://osf.io/k4sh8/ (Accessed June 20, 2022).

[17] Antoniou SA Stabilini C Mavridis D Koutsiouroumpa O Muysoms F. Protocol for EHS Rapid Guideline: Systematic Review, Meta-Analysis, GRADE Assessment, and European Recommendations on Parastomal Hernia Prevention. JAWS (2022) 1:10509. 10.3389/jaws.2022.10509 PMC10831635 38314157

[18] Guyatt GH Oxman AD Kunz R Atkins D Brozek J Vist G GRADE Guidelines: 2. Framing the Question and Deciding on Important Outcomes. J Clin Epidemiol (2011) 64(4):395–400. Epub 2010 Dec 30. PMID: 21194891. 10.1016/j.jclinepi.2010.09.012 21194891

[19] Hultcrantz M Rind D Akl EA Treweek S Mustafa RA Iorio A The GRADE Working Group Clarifies the Construct of Certainty of Evidence. J Clin Epidemiol (2017) 87:4–13. Epub 2017 May 18. PMID: 28529184; PMCID: PMC6542664. 10.1016/j.jclinepi.2017.05.006 28529184 PMC6542664

[20] Tsujimoto Y Fujii T Tsutsumi Y Kataoka Y Tajika A Okada Y Minimal Important Changes in Standard Deviation Units Are Highly Variable and No Universally Applicable Value Can Be Determined. J Clin Epidemiol (2022) 145:92–100. Epub ahead of print. PMID: 35091045. 10.1016/j.jclinepi.2022.01.017 35091045

[21] Tzanis AA Stabilini C Muysoms FE Rossi L Koutsiouroumpa O Mavridis D Update Systematic Review, Meta-Analysis and GRADE Assessment of the Evidence on Parastomal Hernia Prevention—A EHS, ESCP and EAES Collaborative Project. J Abdom Wall Surg (2023) 2:11550. 10.3389/jaws.2023.11550 PMC10831653 38312423

[22] Brandsma HT Hansson BM Aufenacker TJ de Jong N V Engelenburg KC Mahabier C Prophylactic Mesh Placement during Formation of an End-Colostomy: Long-Term Randomized Controlled Trial on Effectiveness and Safety. Ann Surg (2023) 278:e440–e446. Epub ahead of print. PMID: 36727747. 10.1097/SLA.0000000000005801 36727747

[23] Sterne JAC Savović J Page MJ Elbers RG Blencowe NS Boutron I RoB 2: A Revised Tool for Assessing Risk of Bias in Randomised Trials. BMJ (2019) 366:l4898. PMID: 31462531. 10.1136/bmj.l4898 31462531

[24] GRADEpro. GDT: GRADEpro Guideline Development Tool [Software]. McMaster University and Evidence Prime (2022). Available from: https://www.gradepro.org (Accessed January 1, 2022).

[25] Schünemann H Brożek J Guyatt G Oxman A. GRADE Handbook: 5 Quality of Evidence (2022). Available in: https://gdt.gradepro.org/app/handbook/handbook.html#h.9rdbelsnu4iy (Accessed January 1, 2022).

[26] Lambrecht JR Larsen SG Reiertsen O Vaktskjold A Julsrud L Flatmark K. Prophylactic Mesh at End-Colostomy Construction Reduces Parastomal Hernia Rate: A Randomized Trial. Colorectal Dis (2015) 17(10):O191–7. PMID: 26179984. 10.1111/codi.13065 26179984

[27] Näverlo S Gunnarsson U Strigård K Näsvall P. Quality of Life after End Colostomy Without Mesh and With Prophylactic Synthetic Mesh in Sublay Position: One-Year Results of the STOMAMESH Trial. Int J Colorectal Dis (2019) 34(9):1591–9. Epub 2019 Aug 7. PMID: 31392405. 10.1007/s00384-019-03359-2 31392405

[28] Brandsma HT Hansson BM Aufenacker TJ van Geldere D van Lammeren FM Mahabier C Prophylactic Mesh Placement to Prevent Parastomal Hernia, Early Results of a Prospective Multicentre Randomized Trial. Hernia (2016) 20(4):535–41. Epub 2015 Oct 28. PMID: 26511879. 10.1007/s10029-015-1427-9 26511879

[29] Brandsma HT Hansson BM Aufenacker TJ van Geldere D Lammeren FM Mahabier C Prophylactic Mesh Placement During Formation of an End-Colostomy Reduces the Rate of Parastomal Hernia: Short-Term Results of the Dutch PREVENT-Trial. Ann Surg (2017) 265(4):663–9. PMID: 27471840. 10.1097/SLA.0000000000001903 27471840

[30] Jänes A Cengiz Y Israelsson LA. Preventing Parastomal Hernia With a Prosthetic Mesh. Arch Surg (2004) 139(12):1356–8. PMID: 15613293. 10.1001/archsurg.139.12.1356 15613293

[31] Jänes A Cengiz Y Israelsson LA. Preventing Parastomal Hernia With a Prosthetic Mesh: A 5-year Follow-Up of a Randomized Study. World J Surg (2009) 33(1):118–21. PMID: 19011935. 10.1007/s00268-008-9785-4 19011935

[32] López-Cano M Lozoya-Trujillo R Quiroga S Sánchez JL Vallribera F Martí M Use of a Prosthetic Mesh to Prevent Parastomal Hernia During Laparoscopic Abdominoperineal Resection: A Randomized Controlled Trial. Hernia (2012) 16(6):661–7. Epub 2012 Jul 11. PMID: 22782367. 10.1007/s10029-012-0952-z 22782367

[33] López-Cano M Serra-Aracil X Mora L Sánchez-García JL Jiménez-Gómez LM Martí M Preventing Parastomal Hernia Using a Modified Sugarbaker Technique With Composite Mesh During Laparoscopic Abdominoperineal Resection: A Randomized Controlled Trial. Ann Surg (2016) 264(6):923–8. PMID: 27828820. 10.1097/SLA.0000000000001684 27828820

[34] Mäkäräinen-Uhlbäck EJ Klintrup KHB Vierimaa MT Carpelan-Holmström MA Kössi JAO Kairaluoma MV Prospective, Randomized Study on the Use of Prosthetic Mesh to Prevent a Parastomal Hernia in a Permanent Colostomy: Results of a Long-Term Follow-Up. Dis Colon Rectum (2020) 63(5):678–84. PMID: 32032196. 10.1097/DCR.0000000000001599 32032196

[35] Correa Marinez A Bock D Erestam S Engström A Kälebo P Nielsen YW Methods of Colostomy Construction: No Effect on Parastomal Hernia Rate: Results From Stoma-Const-A Randomized Controlled Trial. Ann Surg (2021) 273(4):640–7. PMID: 32209907. 10.1097/SLA.0000000000003843 32209907

[36] Odensten C Strigård K Rutegård J Dahlberg M Ståhle U Gunnarsson U Use of Prophylactic Mesh When Creating a Colostomy Does Not Prevent Parastomal Hernia: A Randomized Controlled Trial-STOMAMESH. Ann Surg (2019) 269(3):427–31. PMID: 29064900; PMCID: PMC6369967. 10.1097/SLA.0000000000002542 29064900 PMC6369967

[37] Pizza F D'Antonio D Lucido FS Del Rio P Dell'Isola C Brusciano L Is Absorbable Mesh Useful in Preventing Parastomal Hernia After Emergency Surgery? The PARTHENOPE Study. Hernia (2022) 26(2):507–16. PMID: 35195798. 10.1007/s10029-022-02579-w 35195798

[38] Prudhomme M Rullier E Lakkis Z Cotte E Panis Y Meunier B End Colostomy With or Without Mesh to Prevent a Parastomal Hernia (GRECCAR 7): A Prospective, Randomized, Double Blinded, Multicentre Trial. Ann Surg (2021) 274(6):928–34. PMID: 33201089. 10.1097/SLA.0000000000004371 33201089

[39] Ringblom C Odensten C Strigård K Gunnarsson U Näsvall P. No Reduction in Parastomal Hernia Rate 3 Years After Stoma Construction With Prophylactic Mesh: Three-Year Follow-Up Results from STOMAMESH-A Multicenter Double-Blind Randomized Controlled Trial. Ann Surg (2022) 277:38–42. Epub ahead of print. PMID: 35837972. 10.1097/SLA.0000000000005537 35837972 PMC9762699

[40] Serra-Aracil X Bombardo-Junca J Moreno-Matias J Darnell A Mora-Lopez L Alcantara-Moral M Randomized, Controlled, Prospective Trial of the Use of a Mesh to Prevent Parastomal Hernia. Ann Surg (2009) 249(4):583–7. PMID: 19300232. 10.1097/SLA.0b013e31819ec809 19300232

[41] Târcoveanu E Vasilescu A Cotea E Vlad N Palaghia M Dănilă N Parastomal Hernias - Clinical Study of Therapeutic Strategies. Chirurgia (Bucur) (2014) 109(2):179–84. PMID: 24742407. 24742407

[42] Vierimaa M Klintrup K Biancari F Victorzon M Carpelan-Holmström M Kössi J Prospective, Randomized Study on the Use of a Prosthetic Mesh for Prevention of Parastomal Hernia of Permanent Colostomy. Dis Colon Rectum (2015) 58(10):943–9. PMID: 26347966. 10.1097/DCR.0000000000000443 26347966

[43] Tiernan J Cook A Geh I George B Magill L Northover J Use of a Modified Delphi Approach to Develop Research Priorities for the Association of Coloproctology of Great Britain and Ireland. Colorectal Dis (2014) 16(12):965–70. PMID: 25284641; PMCID: PMC4262073. 10.1111/codi.12790 25284641 PMC4262073

[44] Burt CG Cima RR Koltun WA Littlejohn CE Ricciardi R Temple LK Developing a Research Agenda for the American Society of Colon and Rectal Surgeons: Results of a delphi Approach. Dis Colon Rectum (2009) 52(5):898–905. PMID: 19502854. 10.1007/DCR.0b013e3181a0b358 19502854

[45] López-Cano M Adell-Trapé M Verdaguer-Tremolosa M Rodrigues-Gonçalves V Badia-Closa J Serra-Aracil X. Parastomal Hernia Prevention With Permanent Mesh in End Colostomy: Failure With Late Follow-Up of Cohorts in Three Randomized Trials. Hernia (2023) 27:657–64. Epub ahead of print. PMID: 36966221. 10.1007/s10029-023-02781-4 36966221 PMC10220116

[46] Findlay JM Wood CPJ Cunningham C. Prophylactic Mesh Reinforcement of Stomas: A Cost-Effectiveness Meta-Analysis of Randomised Controlled Trials. Tech Coloproctol (2018) 22(4):265–70. Epub 2018 May 7. PMID: 29732505; PMCID: PMC5954076. 10.1007/s10151-018-1774-5 29732505 PMC5954076

[47] Mohiuddin S Reeves BC Smart NJ Hollingworth W CIPHER study group. A Semi-markov Model Comparing the Lifetime Cost-Effectiveness of Mesh Prophylaxis to Prevent Parastomal Hernia in Patients Undergoing End Colostomy Creation for Rectal Cancer. Colorectal Dis (2021) 23(11):2967–79. Epub 2021 Aug 12. PMID: 34331840. 10.1111/codi.15848 34331840

[48] Dossa F Baxter NN. Prophylactic Mesh for the Prevention of Parastomal Hernias: Need for a Deep Dive. Ann Surg (2018) 268(2):e29. PMID: 29697456. 10.1097/SLA.0000000000002805 29697456

[49] Odensten C Strigård K Rutegård J Dahlberg M Ståhle U Gunnarson U Response to: “Prophylactic Mesh for the Prevention of Parastomal Hernias: Need for a Deep Dive”. Ann Surg (2018) 268(2):e30. PMID: 29742528. 10.1097/SLA.0000000000002807 29742528

[50] Prudhomme M Fabbro-Peray P Rullier E Occean BV Bertrand MM. Meta-Analysis and Systematic Review of the Use of a Prosthetic Mesh for Prevention of Parastomal Hernia. Ann Surg (2021) 274(1):20–8. PMID: 33378298. 10.1097/SLA.0000000000004704 33378298

[51] Bemelman WA. Prosthetic Mesh for Prevention of Parastomal Hernia: Neither Benefit Nor "Meshed Ups. Ann Surg (2021) 274(1):29–30. PMID: 33856380. 10.1097/SLA.0000000000004895 33856380

[52] Prudhomme M Fabbro-Peray P Occean BV Bertrand MM. Response to the Comment on "Meta-Analysis and Systematic Review of the Use of a Prosthetic Mesh for Prevention of Parastomal Hernia. Ann Surg (2021) 274(6):e912–e913. PMID: 34016816. 10.1097/SLA.0000000000004944 34016816

[53] López-Cano M Pereira JA García-Alamino JM. Comment on: Meta-Analysis and Systematic Review of the Use of a Prosthetic Mesh for Prevention of Parastomal Hernia. Ann Surg (2021) 274(6):e910–e912. PMID: 34029225. 10.1097/SLA.0000000000004949 34029225

