# Supplementary material for: EHS Rapid Guideline: Evidence-Informed European Recommendations on Parastomal Hernia Prevention—With ESCP and EAES Participation
Source: J Abdom Wall Surg. 2023 Sep 14;2:11549. doi: 10.3389/jaws.2023.11549 (PMC10831651; doi:10.3389/jaws.2023.11549)
Supplement: Supplementary file 1 [file DataSheet2.DOCX]

| 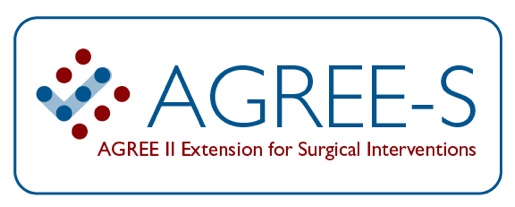 | AGREE-S Reporting Checklist |
| --- | --- |

Title of guideline: EHS Rapid Guideline: Evidence-informed European recommendations on parastomal hernia prevention – with ESCP and EAES participation

Name of first author: Cesare Stabilini

Sponsoring organization (if applicable): European Hernia Society

| **Section** | **Checklist item** | **✔︎** | **Reported in:**  **(page # or manuscript section)** |
| --- | --- | --- | --- |
| Protocol | 1. The guideline has been developed according to a protocol and the link to the protocol is provided. | ✔︎ | Section “Protocol” |
| Objective | 1. The overall objective(s) of the guideline is (are) specifically described. | ✔︎ | Section “Introduction” |
| Health question(s) | 1. The health question(s) covered by the guideline [patient, interventions/procedures, outcomes] are specifically described. | ✔︎ | Section “Health question” |
| Methodological support | 1. The guideline reports on whether it was supported by a guideline development committee, including a guideline methodologist. | ✔︎ | Section “Steering group” |
| Stakeholder involvement | 1. Representation of professional groups and patients included in the guideline development group is reported. | ✔︎ | Section “Guideline panel” |
| Target users | 1. The target users of the guideline are specifically described. | ✔︎ | Section “Health question” |
| Systematic review | 1. The methods that were used to search for evidence are clearly described. | ✔︎ | Section “Systematic review and evidence synthesis” and article published separately |
| Selection criteria | 1. The criteria for selecting the evidence are clearly described. | ✔︎ | Section “Systematic review and evidence synthesis” and article published separately |
| Strengths/limitations of evidence | 1. The strengths and limitations of the body of evidence are clearly described. | ✔︎ | Evidence table (Table 1) |
|  |  |  |  |
| Patient/public input | 1. The views and preferences of the target population (patients, public, etc.) are reported. | ✔︎ | Evidence-to-decision framework (Table 3) |
| Formulation of recommendations | 1. The methods for formulating the recommendations are clearly described. | ✔︎ | Section “Evidence-to-decision framework and development of recommendations” |
| Link between evidence and recommendations | 1. The health benefits, side effects, and risks have been considered in formulating the recommendations. | ✔︎ | Evidence-to-decision framework (Table 3) |
| Link between evidence and recommendations | 1. The link between the recommendations and the supporting evidence is explicitly reported. | ✔︎ | Evidence-to-decision framework (Table 3) |
| Clarity of recommendations | 1. The recommendations are specific and unambiguous. | ✔︎ | Recommendations |
| Alternative options | 1. The different options for management of the condition or health issue are clearly presented. | ✔︎ | Evidence-to-decision framework (Table 3) |
| Identification of recommendations | 1. Key recommendations are easily identifiable. | ✔︎ | Recommendations |
| Resource considerations | 1. The potential resource implications of applying the recommendations have been considered. | ✔︎ | Evidence-to-decision framework (Table 3) |
| Practice variability | 1. The guideline discusses potential variability in surgical expertise of those performing the interventions/procedures. | ✔︎ | Evidence-to-decision framework (Table 3) |
| Role of funder | 1. The role of the funding body is described. | ✔︎ | Funding statement |
| Conflicts of interest | 1. Competing interests of guideline development group members are reported in detail. | ✔︎ | Conflict of interest statement |
| Facilitators and barriers | 1. The guideline describes facilitators and barriers to its application. | ✔︎ | Section “Barriers and facilitators” |
| Update | 1. A procedure for updating the guideline is provided. | ✔︎ | Section “Update” |
| Implementation | 1. The guideline provides advice and/or tools on how the recommendations can be put into practice. | ✔︎ | Section “Barriers and facilitators” |
| Monitoring | 1. The guideline presents monitoring and/or auditing criteria. | ✔︎ | Section “Monitoring” |

Copyright: The GAP Consortium
