# Supplementary material for: EHS Rapid Guideline: Evidence-Informed European Recommendations on Parastomal Hernia Prevention—With ESCP and EAES Participation
Source: J Abdom Wall Surg. 2023 Sep 14;2:11549. doi: 10.3389/jaws.2023.11549 (PMC10831651; doi:10.3389/jaws.2023.11549)
Supplement: Supplementary file 2 [file DataSheet1.DOCX]

| **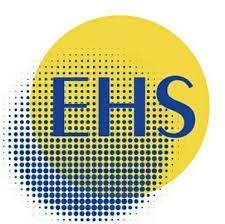** | **EHS Rapid Guideline: Evidence-informed European recommendations on parastomal hernia prevention –**  **with ESCP and EAES participation** | **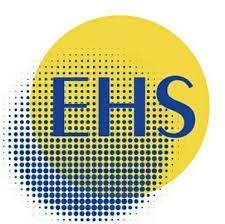** |
| --- | --- | --- |

**Patient version**

This is a patient version of the EHS Rapid Guideline: Evidence-informed European recommendations on parastomal hernia prevention – with ESCP and EAES participation , version 1.2023.

The development of this document complied with the reporting checklist for public versions of guidelines: RIGHT-PVG.

**1 Contact information**

Cesare Stabilini
Secretary of Scientific Advisory Committee
email: [cesarestabil@hotmail.com](mailto:cesarestabil@hotmail.com)

**2 Key points**

- Patients receiving a permanent stoma of the large bowel are at high risk to develop a hernia through the orifice of the stoma, called a parastomal hernia.
- A parastomal hernia may not cause any symptoms, but frequently affects quality of life.
- Reinforcing the abdominal wall with a synthetic material called ‘mesh’ has been postulated to reduce the risk of developing a parastomal hernia, but data are conflicting.
- A systematic review of scientific data suggest that the use of a mesh at least delays the development of a hernia.
- Considering the evidence, cost and resources, feasibility and acceptability issues, and specific input from patient representatives, an interdisciplinary panel suggests the use of mesh for construction of a permanent large bowel stoma, after discussion with the patient about the risks and benefits.
- The panel recommends the routine use of a mesh in patients at higher risk to develop a parastomal hernia, such as patients with a hernia and obesity.

**3 Introduction to the target topic**

Patients with a permanent large bowel stoma have a risk of up to 50% to present with a hernia related to the stoma. A ‘parastomal hernia’ frequently affects quality of life. Placing a mesh within or against the abdominal wall has been suggested to prevent the development of a parastomal hernia.

**4 Purpose, scope and target users**

This document aims to provide information to patients who are scheduled to receive a permanent large bowel stoma. The executive summary is available on the [EHS website](https://www.europeanherniasociety.eu/science), with relative outcomes of a stoma construction with and without a mesh.

**5 Link to the source guideline**

The full guideline is available [here](https://www.europeanherniasociety.eu/science) and published in the [Journal of Abdominal Wall Surgery](https://www.frontierspartnerships.org/journals/journal-of-abdominal-wall-surgery), official journal of EHS.

**6 Recommendations**

- We suggest the use of a synthetic non-absorbable prophylactic mesh for the construction of an end colostomy. (conditional recommendation)
- We recommend the use of a synthetic non-absorbable prophylactic mesh for the construction of an end colostomy in patients at high risk for parastomal hernia (patients with a history of an abdominal wall hernia, connective tissue disorder, obesity, likely to undergo chemotherapy) and life expectancy over 2 years. (strong recommendation)

**7 Questions to ask**

Questions you may want to ask your surgeon:

- Is there any specific reason why I should not have a mesh?
- By how much is my risk to have a parastomal hernia reduced, by using a mesh?
- Is it difficult for you to use a mesh to construct my stoma?
- Does the use of a mesh eliminate the risk of having a parastomal hernia?

**8 Funding**

This project was funded by the European Hernia Society, a not-for-profit organization.

**9 Conflicts of interest**

The steering group, the evidence search group, and the guideline panel members declared no direct (financial) nor indirect (intellectual) conflicts of interest throughout the development of this guideline. Indirect (intellectual) conflicts of external advisors are not considered to have affected the content of this guideline, because they did not have voting rights on the direction and the strength of the recommendations. The funding organization had no influence on the development of this guideline.

**10 Reference**

Wang X, Chen Y, Akl EA, Tokalić R, Marušić A, Qaseem A, Falck-Ytter Y, Lee MS, Siedler M, Barber SL, Zhang M, Chan ESY, Estill J, Kwong JSW, Okumura A, Zhou Q, Yang K, Norris SL; RIGHT working group. The reporting checklist for public versions of guidelines: RIGHT-PVG. Implement Sci. 2021 Jan 11;16(1):10. doi: 10.1186/s13012-020-01066-z. PMID: 33430911; PMCID: PMC7798200.
